# What data and analytics can and do say about effective learning

**DOI:** 10.1038/s41539-017-0006-5

**Published:** 2017-02-09

**Authors:** Jason M. Lodge, Linda Corrin

**Affiliations:** 1 Melbourne Centre for the Study of Higher Education & ARC-SRI Science of Learning Research Centre, University of Melbourne, Melbourne, Australia; 2 Melbourne Centre for the Study of Higher Education, University of Melbourne, Melbourne, Australia

The collection and analysis of data about learning is a trend that is growing exponentially in all levels of education. Data science is poised to have a substantial influence on the understanding of learning in online and blended learning environments. The mass of data already being collected about student learning provides a source of greater insights into student learning that have not previously been available, and therefore is liable to have a substantial impact on and be impacted by the science of learning in the years ahead.^1^

However, despite the potential evident in the application of data science to education, several recent articles, e.g.,^2, 3^ have pointed out that student behavioural data collected en masse do not holistically capture student learning. Rogers^4^ contends that this positivist view of analytics in education is symptomatic of issues in the social sciences more broadly. While there is undeniable merit in bringing a critical perspective to the use of data and analytics, we suggest that the power and intent of data science for understanding learning is now becoming apparent. The intersection of the science of learning with data and analytics enables more sophisticated ways of making meaning to support student learning.

## Learning analytics and the science of learning

The concept of learning analytics emerged less than a decade ago to describe the analysis of student data to inform the improvement of learning and learning environments.^5^ Learning analytics involves the integration and analysis of data from multiple sources to inform action. It is a rapidly growing field that has been built upon a foundation informed by not only by data science but also psychology, business analytics, and the science of learning (see also^6^). Studies of learning analytics have been conducted in areas such as the support of student learning through the provision of automated feedback^7^ and curriculum design.^8^

The strength of learning analytics as a growing field of research and practice is that it leverages the increasing body of data about student behaviour and engagement generated as more technology is used in teaching and learning. The resulting explosion of data has led to many possibilities such as better monitoring of student progress, identification of students “at risk”, new insights into students’ patterns of behaviour, and real-time intervention in digital environments. The use of these data also raises concerns such as ethical use of data, the quality of models underpinning learning analytics systems, and appropriate interpretation of data.^9^ There are several reasons why arguments against the use of these data through learning analytics to understand effective learning are symptomatic of a field still to reach its full potential. We will briefly address these in turn.

## Inferring learning from behaviour

Common criticisms of learning analytics suggest that behavioural data alone cannot be used to determine the quality of learning.^3^ But what is forgotten in these discussions is that the use of behavioural data to understand student learning is far from a novel approach. Researchers working within the science of learning have been using similar inferential methods for decades, particularly psychological scientists and cognitive neuroscientists. Through carefully designed experimental studies, researchers can make inferences about the learning process on the basis of these data. Learning analytics as a methodology can learn from this experience.

What experimental studies provide are models of how learning works that can then be used as a way of understanding and predicting the learning process in real-life settings.^10^ For example, laboratory studies provide suggestions as to the type of behaviours evident when a student gets confused when undertaking a learning activity (e.g., ref. 11). When these same markers become evident when students learn in a real online environment, we can say with some confidence that the student could be confused.

Having identified potential confusion, appropriate educational interventions can be made. This could be an automated feedback message within the online learning system, or some form of communication from/with the teacher. Behavioural data can also be used to track students’ approaches to study. For example, the frequency and sequence with which they engage with learning activities can be tracked.^12^ While this may not directly measure student learning, it can have a positive influence on the student’s learning environment by helping to identify strategies that could improve how they plan and regulate their study.

With some care about the inferences being made on the basis of data, the science of learning and learning analytics can not only learn from each other but could form a fruitful collaboration. Psychological science in particular can provide options for how best to infer learning from behavioural data. Learning analytics provides new tools for the science of learning to assess these options to understand learning in real-life digital environments.

Through collecting, integrating, and analysing data, learning analytics provides opportunities for further examination of how observations from the laboratory translate to the classroom. Log files and audit trails from online learning systems accumulate behavioural data about students as they learn in digital environments that can then be compared and contrasted with behavioural data collected in experimental settings. Learning analytics, and data science more broadly, can therefore help to bridge education, psychology, and neuroscience through a common focus on behaviour.

## Data and design

In the real-life educational environments, creating meaning from data requires making reference to how learning activities are designed.^13^ Similar to the way in which the design of an experiment allows for inferences to be made about learning in the lab, learning design allows for inferences to be made about learning in the classroom. The conditions in both cases give meaning to the data. Examining data about student behaviour with reference to a particular learning design helps teachers to see if students engaged with activities in the way they expected. If not, this might mean the design may need to be reviewed and improved. Again this points to the strength of learning analytics to connect the laboratory and the classroom by bridging student behaviour with learning design.

In conversations about big data there is often an assumption that the data and analysis will automatically provide an “answer” to student learning. The illusion that the collection of big data sets will ultimately lead to conclusive facts about learning is a challenge that the field of learning analytics must address. What is clear at this stage in the development of learning analytics is that the teacher remains central to the process of linking analyses with appropriate educational actions.^14^ As the designer of the learning activities, the teacher is best placed to be able to determine if student patterns of behaviour match with the pedagogical reasons for why that activity should lead to student learning.

## Learning analytics is not just about big data

It is easy to criticise the field of learning analytics as being overly focussed on isolated behavioural markers. It is true that, if this were all that this field represents, there would be limited value in it. However, learning analytics now encompasses a growing range of methods for understanding learning. Its strength is that, when used strategically, it builds on the outcomes of other disciplines, especially research in education and psychology. There is also potential for computational neuroscience to assist in the construction and refining of analytical models that will make better predictions about student learning as they progress.^15^ While learning analytics may not provide the ultimate answer to improving learning, there is potential for the field to help bridge some gaps between education, psychology, and neuroscience by providing deeper insight into student behaviour as they learn in real educational settings.

For learning analytics and behavioural data to be useful for understanding student learning, it is important to determine what we want to know, what is already known, and how this relates to design. Only when these factors are determined, we can identify what data are needed. Identifying the right data is crucial to getting learning analytics right and to realise its potential for bridging the gap between the laboratory and the classroom. Some of these data are easy to access, some may not be available. Some data that are available are not useful. Big data is important, but so too is small data about individuals and particular learning tasks.

The science of learning therefore has a critical role to play in informing how learning analytics evolves over time. Laboratory studies help to verify patterns seen in real-life environments by exposing them to controlled conditions. The science of learning can inform the development of learning analytics through the provision of theories and methodologies that will help to move both fields forward. Learning analytics can help to bridge the gap between neuroscience, psychology, and education by providing a way of observing student behaviour as they learn outside the laboratory. The combination of learning analytics and the science of learning therefore has the potential to provide more powerful ways to monitor and support students as they learn.
